# Phorbol esters seed content and distribution in Latin American provenances of *Jatropha curcas* L.: potential for biopesticide, food and feed

**DOI:** 10.1186/s40064-016-2103-y

**Published:** 2016-04-14

**Authors:** Francisco Bueso, Italo Sosa, Roldan Chun, Renan Pineda

**Affiliations:** Department of Food Science and Technology, EAP Zamorano University, P.O. Box 93, Tegucigalpa, Honduras

**Keywords:** Phorbol esters, *Jatropha curcas*, Cotyledons, Crude oil, Toxic factors, Defatted meal

## Abstract

**Background:**

*Jatropha curcas* L. (Jatropha) is believed to have originated from Mexico and Central America. So far, characterization efforts have focused on Asia, Africa and Mexico. Non-toxic, low phorbol ester (PE) varieties have been found only in Mexico. Differences in PE content in seeds and its structural components, crude oil and cake from Jatropha provenances cultivated in Central and South America were evaluated. Seeds were dehulled, and kernels were separated into tegmen, cotyledons and embryo for PE quantitation by RP-HPLC. Crude oil and cake PE content was also measured.

**Results:**

No phenotypic departures in seed size and structure were observed among Jatropha cultivated in Central and South America compared to provenances from Mexico, Asia and Africa. Cotyledons comprised 96.2–97.5 %, tegmen 1.6–2.4 % and embryo represented 0.9–1.4 % of dehulled kernel. Total PE content of all nine provenances categorized them as toxic. Significant differences in kernel PE content were observed among provenances from Mexico, Central and South America (P < 0.01), being Mexican the highest (7.6 mg/g) and Cabo Verde the lowest (2.57 mg/g). All accessions had >95 % of PEs concentrated in cotyledons, 0.5–3 % in the tegmen and 0.5–1 % in the embryo. Over 60 % of total PE in dehulled kernels accumulated in the crude oil, while 35–40 % remained in the cake after extraction.

**Conclusions:**

Low phenotypic variability in seed physical, structural traits and PE content was observed among provenances from Latin America. Very high-PE provenances with potential as biopesticide were found in Central America. No PE-free, edible Jatropha was found among provenances currently cultivated in Central America and Brazil that could be used for human consumption and feedstock. Furthermore, dehulled kernel structural parts as well as its crude oil and cake contained toxic PE levels.

## Background

*Jatropha curcas* L. (hereafter called Jatropha) is a tropical oilseed plant that probably originated from Mexico and Central America (Edrisi et al. 2015; Ovando-Medina et al. 2011; He et al. 2011). Jatropha was likely disseminated by Portuguese traders to Africa and Asia via Cape Verde and Guinea Bissou Islands (Heller 1996). Re-introduction of non-native cultivars (Cabo Verde) occurred during the late 1990s to Nicaragua (He et al. 2011; Foidl et al. 1996) and later to Honduras and the rest of Central America.

Because of its envisioned industrial and environmental benefits, large scale plantations of Jatropha have been established in Asia (specially India and China), Africa, South America (Colombia and Brazil), Central America (Honduras, Nicaragua, El Salvador and Guatemala) and Mexico (Edrisi et al. 2015). Jatropha plantations (434 ha) have been established in Honduras since 2008 with local and re-introduced materials such as Cabo Verde and India provenances (Puente-Rodriguez 2010). Similar plantation areas have been established in El Salvador and Guatemala with poorly characterized local and imported provenances from India and Mexico.

Individual Jatropha kernel weight range is 0.60–0.85 g. In Latin American provenances, approximately 30–40 % are hulls and 60–70 % seed (Martinez-Herrera et al. 2010; Makkar et al. 1998). Seeds of Jatropha are composed of 97.4 % cotyledons (surrounded and laterally fused to a protein and oil-bearing endosperm), 0.9 % embryo (axis, hypocotyl and epicotyl) and 1.7 % bi-layered cost or tegument (testa and tegmen) (He et al. 2011; Loureiro et al. 2013; Devappa et al. 2012).

Jatropha seeds contain a range of antinutritional compounds such as protease inhibitors (curcin), phytate, lectin, saponines and toxic compounds such as co-carcinogenic phorbol esters (PE) that render its oil and press cake inedible for humans and animals (He et al. 2011; Makkar et al. 1998; Devappa et al. 2012; Haas and Mittelbach 2000). Antinutritional compounds can be eliminated by heat treatment of the cake while PE are not destroyed by roasting (160 °C for 30 min) and migrate to the oil and cake (He et al. 2011; Kumar and Sharma 2008). This is the reason for the classification of toxic and non-toxic genotypes of Jatropha based on PE concentration in seeds (Devappa et al. 2012). Neutralization with NaOH and bleaching during refining reduce 40–60 % PE in crude oil, while degumming and deodorization have very little or no effect (Haas and Mittelbach 2000; Ahmed and Salimon 2009). Alkali and heat treatments have reduced 90 % PE content in whole and dehulled seed meal (Rakshit and Darukeshwara 2008). These reductions, while significant, are not enough to make Jatropha refined oil and meal edible (Goel et al. 2007).

Non-toxic *Jatropha curcas* and *Jatropha platyphylla* have been discovered in Mexico, where they are cultivated and consumed by humans (He et al. 2011; Martinez-Herrera et al. 2010; Makkar et al. 1998, 2011; Devappa et al. 2012). High-PE, toxic Jatropha varieties are cultivated also in Mexico, Central America and the rest of the world (Edrisi et al. 2015; He et al. 2011; Kohli et al. 2009; Gübitz et al. 1999).

Six PE from Jatropha have been characterized (He et al. 2011; Haas et al. 2002). All of them are diterpenoids with a four-ringed tigliane basic skeleton. Separation is made by reverse-phase HPLC (RP-HPLC) with ultraviolet (UV) detection at 280 nm (Makkar et al. 1998) or 240 nm (He et al. 2011) while identification and quantitation is performed with a phorbol-12-myristate 13-acetate internal standard. Most recently, confirmation has been done by LC–MS/MS (Punsuvon et al. 2012).

PE content of toxic Jatropha dehulled seeds from Mexico, Africa and Asia range from 0.5 to 6 mg/g (He et al. 2011; Makkar et al. 1998; Devappa et al. 2012), while non-toxic varieties from Mexico contain up to 0.27 mg/g seed (Martinez-Herrera et al. 2010; Devappa et al. 2012; Makkar et al. 2011; Goel et al. 2007). The seed shell does not contain PE (Devappa et al. 2012). Almost 90 % of PE is present in the storage region of the seed (endosperm-cotyledons), 8–11 % in the coat (mostly in the tegmen) and 0.5 % in the embryo (He et al. 2011; Devappa et al. 2012). After mechanical oil extraction 55–65 % of total seed PE go with the oil while the rest remains in the cake (Saetae and Suntornsuk 2010).

Non-toxic Jatropha provenances have potential for biofuels, food and feeds (Edrisi et al. 2015; Makkar et al. 2011), while high-PE, toxic Jatropha varieties have shown potential as molluscicide (Liu et al. 1997) and insecticide (Kumar and Sharma 2008; Gübitz et al. 1999; Sauerwein et al. 1993) in a variety of crops. The objective of this study was to determine differences in PE content in seeds and its distribution in structural components Jatropha local and re-introduced provenances from Central and South America. Also, to quantify PE content of Jatropha crude oil and cake after oil extraction.

## Results and discussion

### Kernel structure

**Fig. 1:**
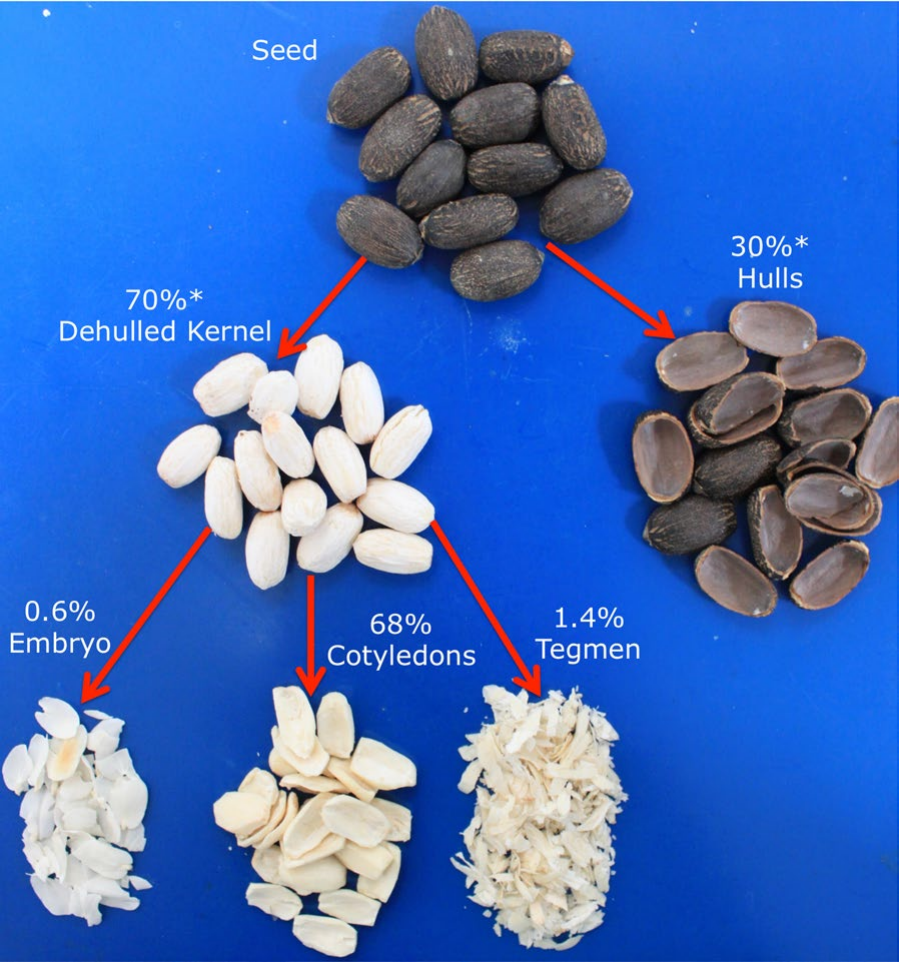
Seed structural parts of *J. curcas* L. Percent of individual structural parts are reported as g with respect to 100 g of seed with hulls

Among the Jatropha provenances from Central, South and North America individual seed weight ranged 0.75–0.91 g. From the whole seed, 27–33 % corresponded to hulls and 67–73 % to dehulled kernel (Fig. 1). Low variability (no significant difference) was observed on proportions of kernel structural parts among provenances from different regions of Latin America. These results are in accordance with seed dimensions and structure previously reported on provenances from Mexico, Nicaragua, Asia and Africa (Martinez-Herrera et al. 2010; Makkar et al. 1998).

Proportions of dehulled Jatropha kernel structural components varied significantly among accessions, while not among country or region of origin (Table 1). Cotyledons comprised 96.2–97.5 %, tegmen 1.6–2.4 % and embryo represented 0.9–1.4 % of the dehulled kernel. Therefore, no phenotypic departures in seed size and structure were observed among Jatropha provenances cultivated in Central, North and South America compared to provenances from Mexico, Asia and Africa (He et al. 2011; Loureiro et al. 2013; Devappa et al. 2012). This might be further indication that currently, the most cultivated Jatropha varieties in the region have a homogeneous, narrow genetic base and are in fact re-introduced non-native cultivars or their progenies (He et al. 2011; Foidl et al. 1996). It has been well documented (Edrisi et al. 2015; He et al. 2011; Foidl et al. 1996; Martinez-Herrera et al. 2010; Kohli et al. 2009) that efforts in the region, have used so far very little of the genetic variability available especially in Mexico, believed to be the center of origin of Jatropha (Edrisi et al. 2015; Ovando-Medina et al. 2011; He et al. 2011).

**Table 1:** Yield of structural parts from dehulled kernel of nine provenances of *J. curcas* L.

Country	Provenance	Cotyledons	Tegmen	Embryo
		(g part/100 g dehulled kernel)		
		Mean ± SD	Mean ± SD	Mean ± SD
Mexico	Mexicana	96.5^cd^ ± 0.4	2.4ª ± 0.3	1.1^ab^ ± 0.1
	Puebla	96.8^bcd^ ± 0.1	2.1^ab^ ± 0.1	1.1^ab^ ± 0.1
El Salvador	Criolla Salvadoreña	97.5^a^ ± 0.2	1.7^cd^ ± 0.2	0.9^b^ ± 0.1
	India Salvadoreña	97.5^ab^ ± 0.2	1.6^c^ ± 0.1	1.0^b^ ± 0.1
Brazil	Embrapa	97.1^abc^ ± 0.3	2.0^abc^ ± 0.2	0.9^b^ ± 0.1
	Bravo × Mali	96.2^d^ ± 0.4	2.4ª ± 0.3	1.4^a^ ± 0.2
Honduras	111	96.6^bcd^ ± 0.3	2.1^ab^ ± 0.2	1.2^ab^ ± 0.1
	Arturo Araujo	97.1^abc^ ± 0.2	1.9^abc^ ± 0.1	1.0^b^ ± 0.1
Nicaragua	Cabo Verde	97.3^abc^ ± 0.2	2.0^abc^ ± 0.2	0.7b ± 0.1
%CV		0.3	9.5	11.7

Data are from nine provenances from five Latin American countries and three seed structural parts. Means with different superscript letters on the same column are significantly different (LSD test, P < 0.05)

*SD* standard deviation, *%CV* percent coefficient variation

### Phorbol ester distribution

**Fig. 2:**
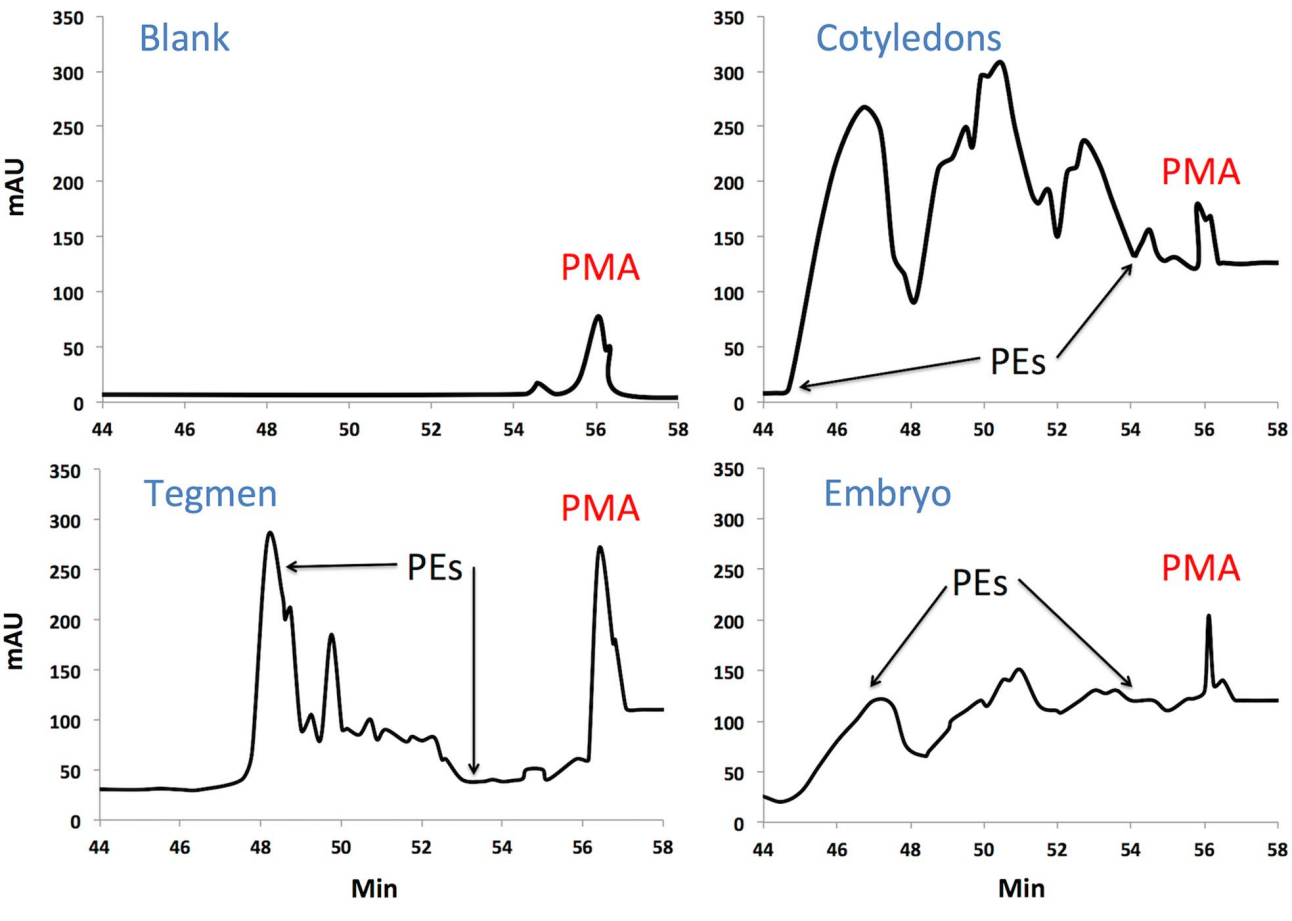
Chromatograms of phorbol esters from *J. curcas* dehulled kernel structural parts. *PMA* phorbol 12-myristate, 13-acetate, *PE* phorbol esters

Seven peaks corresponding to PE were identified between min 44 and 54 (Fig. 2) as previously reported (He et al. 2011). Most of PE concentrated in dehulled seed as previously reported (Saetae and Suntornsuk 2010). PE content of hulls was low (1–8 % of total PE), ranging from 0.10 mg/g (Mexican) to 0.20 mg/g (Cabo Verde). Significant differences in dehulled kernel PE content were observed among provenances from Mexico, Central and South America (P < 0.01), being Mexican the highest (7.6 mg/g) and Cabo Verde the lowest (2.57 mg/g). However, no regional trend was detected in total PE content within the kernel during the pre-screening of the germoplasm collection or in this study (Table 2). Results were similar to PE content of toxic Jatropha dehulled seeds from Mexico, Africa and Asia range from 0.5 to 6 mg/g (He et al. 2011; Makkar et al. 1998; Devappa et al. 2012). Again, this suggests narrow genetic variability both in kernel structure and PE content among currently cultivated cultivars in the region. Non-toxic varieties from Mexico contain up to 0.27 mg/g seed (Martinez-Herrera et al. 2010; Devappa et al. 2012; Makkar et al. 2011; Goel et al. 2007). Therefore, total PE content of provenances fell within the range to be considered as toxic.

**Table 2:** Phorbol ester content of dehulled kernel and structural parts of nine provenances of *J. curcas* L.

Country	Provenance	Dehulled Kernel	Cotyledons	Tegmen	Embryo
		(mg/g kernel)		(mg/g kernel part)	
		Mean ± SD	Mean ± SD	Mean ± SD	Mean ± SD
Mexico	Mexicana	7.56^a^ ± 0.4	7.71ª ± 0.4	2.76^b^ ± 0.2	4.84^a^ ± 0.2
	Puebla	3.15^d^ ± 0.1	3.09^c^ ± 0.1	4.97^a^ ± 0.4	5.33^a^ ± 0.1
El Salvador	Criolla Salvadoreña	3.78^cd^ ± 0.4	3.79^c^ ± 0.4	3.07^b^ ± 0.4	4.10^b^ ± 0.1
	India Salvadoreña	7.36^a^ ± 0.2	7.48ª ± 0.2	2.40^b^ ± 0.1	3.69^b^ ± 0.1
Brazil	Embrapa	4.68^c^ ± 0.6	4.75^bc^ ± 0.6	1.97^b^ ± 0.2	2.69^c^ ± 0.1
	Bravo × Mali	5.72^b^ ± 0.7	5.84^b^ ± 0.7	1.63^c^ ± 0.1	4.05^b^ ± 0.4
Honduras	111	4.18^cd^ ± 0.4	4.22^bc^ ± 0.1	2.11^b^ ± 0.2	4.34^b^ ± 0.1
	Arturo Araujo	3.65^d^ ± 0.2	3.65^c^ ± 0.2	3.10^b^ ± 0.2	4.28^b^ ± 0.3
Nicaragua	Cabo Verde	2.57^e^ ± 0.2	2.64^d^ ± 0.2	2.35^b^ ± 0.3	1.67^d^ ± 0.2
%CV		6.8	7.7	9.2	5.1

PE content is from nine provenances from five Latin American countries and three seed structural parts. Means with different superscript letters on the same column are significantly different (LSD test, P < 0.05)

*SD* standard deviation, *%CV* percent coefficient variation

Variability in PE content among seed structural parts was eight times higher than among accessions from Central, North and South America. This is consistent with inter-regional low phenotypic variability observed in kernel structure and total PE content among provenances in this study. PE was detected in all dehulled seed structural parts from all accessions (Fig. 2). All accessions had 95–99 % of PEs concentrated in cotyledons and only 0.5–3 % in the tegmen and 0.5–1 % in the embryo, similar to previously reported PE distribution in kernel parts (He et al. 2011; Devappa et al. 2012). Less PE content was found in tegmen than on cultivars from other regions. However, this might be due to internal migration to the cotyledon and embryo (He et al. 2011). Cotyledons had the highest concentration of PEs per g, while tegmen had the lowest in most provenances (Table 2). This discards the possibility of mechanically detoxifying Jatropha seeds by removing the tegmen to make them edible. No tendency was observed between origin of provenances and total PE content or content per kernel structural part.

### Dehulled kernel toxicity

Table 2 shows all structural parts from all nine accessions were considered toxic due to its PE content (>0.27 mg PE/g) (Martinez-Herrera et al. 2010; Devappa et al. 2012; Makkar et al. 2011; Goel et al. 2007). That limits their use beyond biodiesel to non-food or feed applications. Non-toxic Jatropha varieties have been found in south and western Mexico (Makkar et al. 1998), opening the door for production of improved edible Jatropha varieties/hybrids in the near future. No edible Jatropha provenances have been found outside Mexico so far, and none were found in this study or in the pre-screening of the 118-entry germoplasm collection from Latin America.

Gene mapping and breeding assisted with genetic engineering efforts to produce Jatropha cultivars that combine agronomic performance with high edible oil/cake yields are incipient in the region and worldwide (Edrisi et al. 2015; He et al. 2011). There are sufficient examples of toxic oilseeds (high-gossypol cotton, high-erucic acid rapeseed) and wild, toxic marginal-land crops (high-tannin sorghum) that were transformed into industrial food crops through traditional breeding, once non-toxic accessions were discovered in their respective centers or origin. Efforts aiming to transform Jatropha into an industrial food and biofuel crop (Edrisi et al. 2015; Ovando-Medina et al. 2011; Kohli et al. 2009) should follow the same path and take advantage of new genetic engineering tools to speed up the process. High-PE provenances (Mexicana, India Salvadoreña), have potential applications as bio-pesticides and should be bred separately for that purpose. High-PE, toxic Jatropha varieties have shown potential as molluscicide (Liu et al. 1997) and insecticide (Kumar and Sharma 2008; Gübitz et al. 1999; Sauerwein et al. 1993) in a variety of crops.

### Phorbol esters in oil and cake

After mechanical oil extraction of Salvadorian and Nicaraguan provenances, 60–65 % of total PE in dehulled kernels accumulated in the crude oil, while 35–40 % remained in the cake (Table 3). Previous studies have reported that after mechanical oil extraction, 55–65 % of total seed PE go with the oil while the rest remains in the cake (Saetae and Suntornsuk 2010). Both crude oil and cake can therefore be considered as toxic due to remaining PE content.

**Table 3:** Phorbol ester content of seed, oil and defatted meal of three provenances of *J. curcas* L. PE content is from dehulled seeds, crude oil and untreated seed cake after mechanical extraction

Variety	Seed	Oil	Cake
	(mg PE/g kernel)		
Mexicana	7.56^a^	4.91^a^	3.25^a^
India Salvadoreña	7.36^a^	4.63^a^	3.02^a^
Cabo Verde	2.57^b^	1.67^b^	0.55^b^

Means with different superscript letters on the same column are significantly different (LSD test, P < 0.05)

## Conclusions

Low phenotypic variability in seed physical, structural traits and PE content was observed among provenances from Latin America. Very high-PE provenances with potential as biopesticide were found in Central America. No PE-free, edible Jatropha was found among provenances currently cultivated in Central America and Brazil that could be used for human consumption and feedstock. Furthermore, dehulled kernel structural parts as well as its crude oil and cake contained toxic PE levels.

## Experimental

### Plant materials

Nine Jatropha varieties (Table 4) from Honduras (Arturo Araujo and 111), Brazil (Bravo × Mali and EMBRAPA), El Salvador (Criolla Salvadoreña and India Salvadoreña), Nicaragua (Cabo Verde) and Mexico (Mexican and Puebla) were selected from 118 pre-screened provenances (because of their wide use, superior agronomic performance and oil content within country/region of origin) from the germplasm collection planted at EAP Zamorano University, Honduras on 2010. Provenances were planted at the Monte Redondo Experimental Station on slightly acid (pH 6.1), loamy soil (44 % sand, 36 % silt and 20 % clay) with 1.9 % organic matter. The plantation was maintained under drip irrigation during the dry season (December–April) and the summer heat wave (July 15th–August 15th) as needed to avoid stress. A pruning to stimulate growth took place in November, while two more prunings for pest control and quality of seed were done during the dry season. Fertilization with N–P–K (20–20–20) formula was applied at the beginning of the rainy season (May) and complemented with foliar fertilization at pre-flowering and flowering stages. Fertilizations were based on Jatropha requirements for optimum seed production and soil analysis (low total nitrogen, extractable phosphorus and potassium). Mature seed samples were obtained on three harvest dates (July 2012, July and August 2013) and dried to 13–14 % moisture before processing.

**Table 4:** Origin of nine *J. curcas* L. provenances

Provenance	Country	Town	Latitude	Longitude
Mexicana	Mexico	Poza Rica de Hidalgo, Veracruz	20°07′04″N	97°33′35″W
Puebla	Mexico	Huitzilan, Puebla	20°01′03.1″N	97°41′45.1″W
Criolla Salvadoreña	El Salvador	San Julian, Sonsonate	13°37′38.4″N	89°47′10.6″W
India Salvadoreña	El Salvador	Jocoro, Morazan	13°35′23.3″N	88°04′23.3″W
Embrapa	Brazil	Brasilia	15°43′55.7″S	47°54′00.6″W
Bravo × Mali	Brazil	Belo Horizonte, Minas Gerais	19°52′08.6″S	43°57′57.3″W
111	Honduras	San Antonio de Oriente, Francisco Morazan	14°00′32.6″N	86°59′13.0″W
Arturo Araujo	Honduras	Sulaco, Yoro	14°54′49.9″N	87°15′51.2″W
Cabo Verde	Nicaragua	Rivas, Rivas	11°26′34.3″N	85°48′41.2″W

Data and GPS coordinates are from nine provenances from five Latin American countries

### Chemicals

Phorbol 12-myristate, 13-acetate (PMA) was purchased from Sigma-Aldrich Co. (St. Louis, MO, USA); acetonitrile and methanol Lichrosolv HPLC grade, from Merck (Guatemala, Guatemala); hexane and isopropanol Chromasolv HPLC grade, from Sigma-Aldrich Co. (St. Louis, MO, USA). All other chemicals were of reagent grade.

### Sample preparation for analysis

The seeds were manually cracked to separate shells and kernels and 100 g samples of dehulled kernels were taken as experimental units. Kernel percent moisture (g/100 g) was measured by AOAC 952.08 method (convection oven). Structural parts (tegmen, cotyledons and embryo) were separated manually under a magnifying lens with scalpel and forceps and weighed with an analytical balance to the nearest 0.1 mg (Fig. 1). Cotyledons and embryo were powdered with mortar and pestle, while tegmen was reduced with a cyclon mill prior to analysis.

### Oil extraction

Jatropha seeds were dehulled with a DME-100 dehuller (Ecirtec, Bauru, Brazil) at 20 % fan speed. Jatropha oil was extracted from dehulled seeds with an Ecirtec MPE-40 stainless steel expeller at 30 % screw power and 1 cm opening. Crude oil was filtered by pumping through an Ecirtec FPE-20/10 TI press-filter using a configuration of ten stainless steel plates and 11 cloth frames (20 × 20 cm). Filtered oil was degummed in an Ecirtec 25 kg open stainless steel reactor with 0.2 % citric acid and 3 % water by weight of the oil at 90 °C under continuous agitation for 60 min. The gum-water phase was precipitated and subsequently removed by gravity from the reactor (Kumar and Sharma 2008). Defatted cake and degummed oil were collected and stored at 4 °C until analysis.

### Phorbol ester analysis

300 mg of sample and 50 μg PMA (internal standard) were mixed and extracted with 4 ml of a hexane–isopropanol (3:2) solution. The extract was disolved in acetonitrile, defatted with hexane, passed through a 0.45 μm polytetrafluoroethylene (PTFE) filter and concentrated under a stream of N_2_ to 300 μl in vials for HPLC injection (He et al. 2011).

PE and PMA were identified and quantified (mg PE/g sample, dry basis) with an Agilent Eclipse Plus C_18_ column (150 mm × 4.6 mm × 5 μm) by HPLC–DAD at 240 nm (He et al. 2011).

### Statistical analysis

A split plot design with three replicates (harvest date) was used. The main plots were nine accessions and the subplots were three dehulled seed structural parts (tegmen, cotyledons and embryo).

Statistical analysis of results was performed with SAS v. 9.3 (SAS Institute, Cary, NC, USA). The Proc GLM procedure was used for performing analysis of variance (ANOVA), followed by LSD means separation test when no significant provenance × kernel part interaction was found. The LS means procedure was employed when provenance × kernel part interaction was significant.

## Abbreviations

Jatropha: *Jatropha curcas* L.
PE: phorbol esters
RP-HPLC: reverse phase high performance liquid chromatography
UV: ultraviolet
LC–MS/MS: liquid chromatography with tandem mass spectrometry
PMA: phorbol 12-myristate, 13-acetate
%CV: percent coefficient of variation
SD: standard deviation
PTFE: polytetrafluoroethylene
HPLC–DAD: high performance liquid chromatography with diode array detector
ANOVA: analysis of variance
